# The Effect of Reduction Mammaplasty on the Vertebral Column: A Radiologic Study

**DOI:** 10.1155/2013/701391

**Published:** 2013-08-25

**Authors:** Onder Karaaslan, H. Gokhan Demirkiran, Ozlem Silistreli, Erhan Sonmez, Yagmur Kaan Bedir, Melih Can, Gorkem Caliskan, Cem Aslan, Meltem Ayhan Oral, Yuksel Kankaya

**Affiliations:** ^1^Plastic Reconstructive and Aesthetic Surgery Clinic, Ataturk Training and Research Hospital, Izmir Katip Celebi University, 35360 Izmir, Turkey; ^2^Orthopedic Surgery Department, Hacettepe University Faculty of Medicine, 06100 Ankara, Turkey; ^3^Plastic Reconstructive and Aesthetic Surgery Clinic, Ankara Training and Research Hospital, 06340 Ankara, Turkey

## Abstract

Some studies emphasized that anatomic mechanisms of vertebral aberrations could be associated with large breasts. The effect of mammaplasty operation on the vertebral column and body posture seems to be beneficial; in this trial, it was planned to investigate the objective radiologic effect of reduction mammaplasty on the posture of the vertebral column in a group of patients operated due to the large breasts. Thirty-four white women with large breasts were enrolled in this study. The patients were divided into three groups according to their breast cup sizes. Anteroposterior and lateral radiographs of the lumbosacral and thoracic spine were taken at baseline preoperatively, and the same radiographic images were taken in an average of 12 months later than the reduction mammaplasty operation. All were evaluated and compared for thoracic kyphosis angle and lumbar lordosis angle both preoperatively and postoperatively. The mean thoracic kyphosis angle was 40,53 preoperatively and 39,38 postoperatively. However, there was no statistically significant difference between the preoperative and postoperative measurements in all groups (*P* > 0,05). The mean lumbar lordosis angle was 54,71 preoperatively and 53,18 postoperatively. Regarding the preoperative and postoperative measurements of lumbar lordosis angles, no statistically significant difference was found between the groups (*P* > 0,05). Although breast size may be an important factor that affects body posture, reduction mammaplasty operations have little or no radiologic effect on the vertebral column.

## 1. Introduction

Hypertrophic large breasts are common and may be causative factor for impairment of spine function. The most common symptoms of women suffering from hypertrophic breasts are malposture, painful shoulder grooves, persistent submammary intertrigo, and back pain [1, 2]. Reduction mammaplasty is a surgical option performed to reduce weight and reshape figures in symptomatic women. Breast reduction surgery is commonly believed to reduce shoulder, neck, and upper back pain; improve body image and posture; improve shoulder and back function; and prevent persistent submammary dermatoses [3]. The other important difficulties related to the large size of breasts are finding suitable clothes and uncomfortable feelings in body image and sexual relationships [4]. The positive effects of reduction mammaplasty have been documented in several, mostly retrospective, studies [4–8]. Despite the complex health burden for women patients with hypertrophic breasts, there has been controversy regarding whether breast reduction is a functional or an aesthetic procedure.

In a study, it was documented that breast size seems to be an important factor that affects body posture, especially the thoracic kyphosis and lumbar lordosis angles [9]. The results of this study made us plan a survey to evaluate the postural change of the vertebral column in a group of patients operated due to the breast hypertrophy. We designed a radiologic study to examine and compare postural aberrations of the back and lower back regions before and after the breast reduction.

## 2. Materials and Methods

Thirty-four patients with breast hypertrophy were referred to our clinic for evaluation and appropriate therapy in the period of September 2010 and March 2011. We received approval by the Local Hospital Ethical Committee and also obtained the informed consent from the patients before study. The patients having at least one of the following criteria were excluded from the study: (1) systemic or vertebral disease who had undergone spinal surgery, (2) using any medication (3) patients <18 years of age or postmenopausal patients (because postmenopausal osteoporosis is a main cause of the postural aberrations) [9, 10] and (4) pregnant women (possible X-ray damage to the fetus and spinal alignment changes during the pregnancy) [9, 11].

Age, weight, height, and preoperative cup sizes of the breasts and weight of resected tissues were recorded for every patient. Thirty-four patients were divided into three groups according to the preoperative breast cup sizes—Group I: C cup size (*n* = 10), Group II: D cup size (*n* = 11), and Group III: DD cup size (*n* = 13). There was no patient with A or B breast cup size. For radiologic evaluation, preoperatively anteroposterior and lateral radiographs of the thoracic and lumbosacral regions were obtained with the patients in a relaxed upright standing position without shoes. Body mass index (BMI) was calculated for each patient with using the following formula: weight/height^2^. The cup sizes of the breasts for every patient by calculating the difference between the overbust and underbund measurements are as follows: <6,5 cm (A cup), 6,5–13 cm (B cup), 13–19 cm (C cup), 19,5–26 cm (D cup), and >26 cm (DD cup).

**Figure 1:**
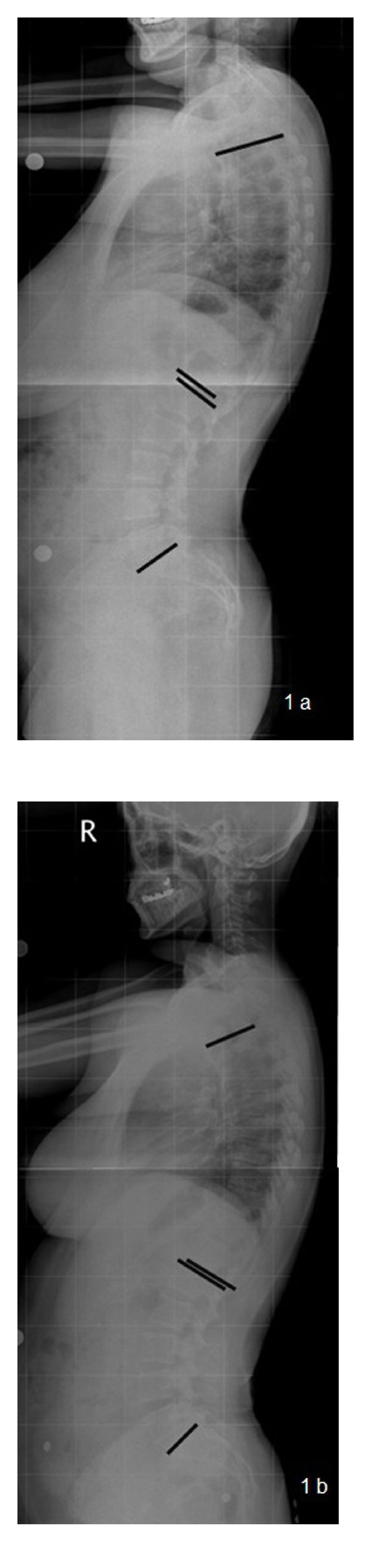
(a) Preoperative lateral X-ray of a 38-year-old patient. The black lines are the landmarks for the measurements of thoracic kyphosis and lumbar lordosis angles. (b) Postoperative (14 months) lateral X-ray of the same patient and used vertebral landmarks for measurements.

Anteroposterior and lateral radiographs of the lumbosacral and thoracic spine were taken at baseline preoperatively and the same radiographic images were taken an average of 12 months later than the reduction mammaplasy operation. On the lateral X-rays, thoracic kyphosis angle (the angle between the planes of superior end plate of the first thoracic vertebra and the inferior end plate of the 12th thoracic vertebra) and the lumbar lordosis angle (the angle between the superior end plate of the first lumbar vertebra and the superior line of the sacrum) were measured by using Cobb's method [12] (Figures 1(a) and 1(b)). A single orthopedic surgeon performed all measurements.

For the statistical analyses of differences between three groups, Kruskal Wallis, Mann-Whitney *U* test, and Pearson correlation analysis by using computerized statistical software (SPSS 16.0 for Windows, SPSS Inc., Chicago, USA) were used. All results are presented as means, ranges, and standard deviations. Differences between the groups in terms of these angles and clinical parameters such as age, BMI, and resected tissue volumes were analyzed and compared.

## 3. Results

We performed a study of 34 women with various breast sizes. The mean age was 33,7 years (range 23–48, SD ± 6,14). The age distribution was homogenous between the groups and there was not statistically significant difference (*P* > 0,05).

The breast cup size was C in 10 patients (Group I), D in 11 patients (Group II), and DD in 13 patients (Group III). For Groups I, II, and III, a balanced representation of women with respect to breast cup size (C, D, and DD) was observed.

**Figure 2:**
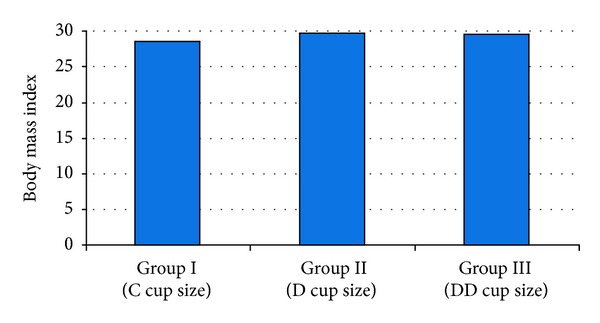
The body mass index (BMI) distribution between the breast cup size groups.

The mean BMI for all women was 29,32 ± 2,24 kg/m^2^ (range 26–34). There was no statistically significant difference between the three groups in terms of BMI (*P* > 0,05) (Figure 2).

**Figure 3:**
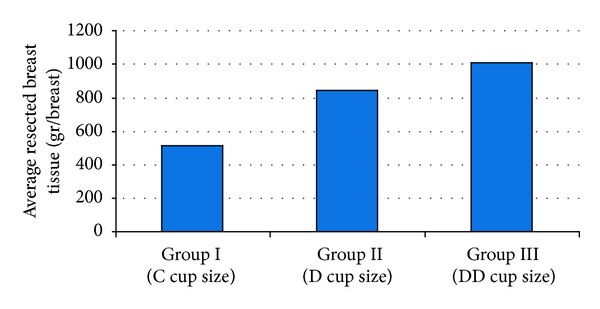
Distribution of resected breast tissue volume (gr/breast) between the cup size groups.

All of the breast reductions were based on a Wise pattern and an inferior pedicle. On average 807,2 gr (SD ± 287, 1; range 310 to 1454) of tissue was resected per breast. There was a statistically significant difference between the groups in terms of resected tissue volumes, as it was significantly lower in Group I (*P* < 0,05); however, there was no significant difference between Groups II and III (*P* > 0,05) (Figure 3).

**Figure 4:**
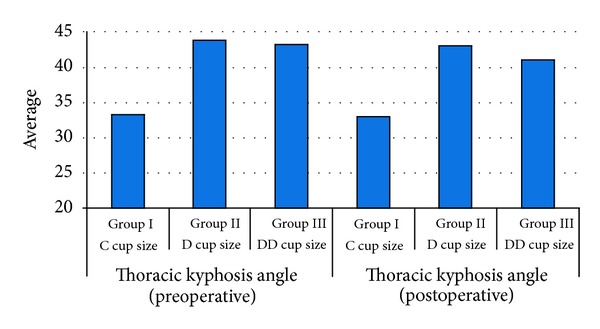
The preoperative and postoperative lumbar lordosis angles distribution between the cup size groups.

The mean thoracic kyphosis angle was 40,53° ± 6,3° (range 25°–52°) preoperatively and 39,38° ± 5,35° (range 25°–49°) postoperatively. Regarding the preoperative and postoperative thoracic kyphosis angles, the lowest score was found in Group I, and it was statistically significant (*P* < 0,05). However, there was no significant difference between the preoperative and postoperative measurements in all groups (*P* > 0,05) (Figure 4).

**Figure 5:**
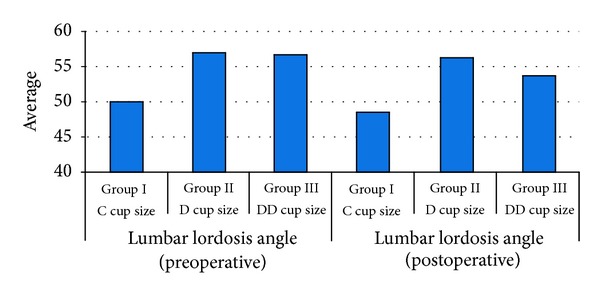
Distribution of the preoperative and postoperative lumbar lordosis angles between the cup size groups.

The mean lumbar lordosis angle was 54,71° ± 7,77° (range 43°–70°) preoperatively and 53,18° ± 7,95° (range 40°–70°) postoperatively. Regarding the preoperative and postoperative measurements of lumbar lordosis angles, there was no significant difference between the groups (*P* > 0,05) (Figure 5).

Overall, the complication rate was low. Delayed wound healing was observed in 2 patients, fat necrosis was observed in one, and postoperative infection developed in one patient.

## 4. Discussion

Malposture, painful shoulder grooves, submammary intertrigo, and back pain are the most common symptoms of women suffering from breast hypertrophy [2]. Some authors have reported that upper extremity neurological symptoms are caused by excessively large breast size, such as ulnar nerve paresthesias, hand numbness, and carpal tunnel syndrome [13–15].

In women presenting with reduction surgery, nonsurgical approaches including weight loss, physical therapy, special brassieres, and medications were not effective in providing permanent relief of macromastia-related symptoms [16]. The main reason for willing this surgery was physical complaints rather than aesthetic appearance. The women in this study did not seem to be very concerned about their body image and this is consistent with other previous reports that show that majority of patients seek breast reduction for noncosmetic reasons [17]. In our patients, a significant amount of tissue was resected (median, 807 g from each breast). Only minor complications were seen; however, bilateral suction drainage was used in whole patients.

It was clearly documented that breast reduction mammaplasty is a good treatment for reducing symptoms of macromastia [18]. Deformities of the vertebral column develop along planes of stress and pull from the center of gravity, causing a kyphotic or increased forward posture [19]. Foreman et al. previously showed that women who underwent reduction mammaplasty surgery demonstrated a 35 percent decrease in low back compressive forces [20]. The previous researchers also demonstrated that higher mechanical loading of the spine increases the rate of disc degeneration and the occurrence of low back disorders [21, 22]. The effect of operation on the vertebral column and body posture seems to be beneficial; we have designed this prospective study to investigate possible radiologic effect on the vertebral column.

In a cohort study, Findikcioglu et al. emphasized that breast size seems to be an important factor that affects body posture, especially the thoracic kyphosis and lumbar lordosis angles [9]. Large breasts have statistically significant physical effects on the vertebral column and may alter the thoracic kyphosis and lumbar lordosis angles [9]. Overweight causes forward leaning from hip and increases lumbar lordosis. Increased lordosis compensated with increases at thoracic kyphosis. The aim of the present study was to address the question of radiologic changes in particular. In present study, D and DD cup size patients have higher lordosis and thoracic kyphosis angles which are not in pathological ranges of sagittal plain. Although breast size is an important factor that affects body posture, data obtained from this radiologic study shows that reduction mammaplasty operations seem to have little or no radiologic effect on the vertebral column, especially in terms of thoracic kyphosis and lumbar lordosis angles.

To the best of our knowledge, this is the second study quantitatively documenting the effect of breast reductions on the spinal posture. In a recently published paper, Findikcioglu et al. studied the same subject and showed that breast reduction surgery causes significant decrease in angles including thoracic kyphosis, lumbar lordosis, and sacral inclination [23]. Although we have used the same methodology and also we have longer follow-up period, our results are totally different. The parameters including the age, BMI, breast cup size, or resected tissue volume may be the causative factors for this controversy. However, there are some limitations about the study that are worthy of mention. We examined a small group including 34 individuals, and postoperative measurements were performed approximately one year later than the operations, and therefore, the generalizability of our findings may be limited. Because, in sagittal plain, postural changes which caused by anterior overweight of breast was acquired in long-term period. Our patients have no changes in lumbar lordosis and thoracic kyphosis after reduction mammaplasty in a short-term period which is average of 12 months in this study. Maybe, further researches with more patients and long-term postoperative surveys or addition of more detailed imaging studies such as magnetic resonance may give more reliable results. Furthermore, body habitus of the patients or muscular contributions that affect posture of the spine were neglected, so additional researches including more comprehensive models and parameters may be designed. Also women who underwent augmentation mammaplasty may be evaluated with spinal X-rays to see an expected opposite change in posture, which may be the subject of an upcoming study, so we are planning another study comparing the postural changes of the spinal angle of the patients who had undergone breast augmentation.

## 5. Conclusion

Reduction mammaplasty is still one of the operations with the highest patient satisfaction. Although this radiologic study shows that reduction mammaplasty operations seem to have little or no radiologic effect, we still believe that this surgery is a medical necessity for patients with large breasts.
